# White-nose syndrome is associated with increased replication of a naturally persisting coronaviruses in bats

**DOI:** 10.1038/s41598-018-33975-x

**Published:** 2018-10-19

**Authors:** Christina M. Davy, Michael E. Donaldson, Sonu Subudhi, Noreen Rapin, Lisa Warnecke, James M. Turner, Trent K. Bollinger, Christopher J. Kyle, Nicole A. S.-Y. Dorville, Emma L. Kunkel, Kaleigh J. O. Norquay, Yvonne A. Dzal, Craig K. R. Willis, Vikram Misra

**Affiliations:** 1 Environmental and Life Sciences Graduate Program, Trent University, Peterborough, ON Canada; 2 Ontario Ministry of Natural Resources and Forestry, Wildlife Research and Monitoring Section, Trent University, Peterborough, ON Canada; 3 Department of Microbiology, Western College of Veterinary Medicine, University of Saskatchewan, Saskatoon, Saskatchewan Canada; 4 Department of Biology, University of Winnipeg, Manitoba, Canada; 5 Present Address: Department of Animal Ecology and Conservation, University Hamburg, Hamburg, Hamburg, Germany; 6 Present Address: Institute for Land Water and Society, Charles Sturt University, Albury, New South Wales Australia; 7 Canadian Wildlife Health Cooperative and Department of Pathology, Western College of Veterinary Medicine, University of Saskatchewan, Saskatoon, Saskatchewan Canada; 8 Forensic Science Department, Trent University, Peterborough, ON Canada

## Abstract

Spillover of viruses from bats to other animals may be associated with increased contact between them, as well as increased shedding of viruses by bats. Here, we tested the prediction that little brown bats (*Myotis lucifugus*) co-infected with the *M. lucifugus* coronavirus (*Myl*-CoV) and with *Pseudogymnoascus destructans* (*Pd*), the fungus that causes bat white-nose syndrome (WNS), exhibit different disease severity, viral shedding and molecular responses than bats infected with only *Myl*-CoV or only *P. destructans*. We took advantage of the natural persistence of *Myl*-CoV in bats that were experimentally inoculated with *P. destructans* in a previous study. Here, we show that the intestines of virus-infected bats that were also infected with fungus contained on average 60-fold more viral RNA than bats with virus alone. Increased viral RNA in the intestines correlated with the severity of fungus-related pathology. Additionally, the intestines of bats infected with fungus exhibited different expression of mitogen-activated protein kinase pathway and cytokine related transcripts, irrespective of viral presence. Levels of coronavirus antibodies were also higher in fungal-infected bats. Our results suggest that the systemic effects of WNS may down-regulate anti-viral responses in bats persistently infected with *M. lucifugus* coronavirus and increase the potential of virus shedding.

## Introduction

Bats are hosts for many viruses and are thought to be the source of some viruses that have spilled over to humans and other mammals, causing fatal disease. These include coronaviruses causing severe acute respiratory syndrome (SARS^1^), Middle East respiratory syndrome (MERS^2–5^), porcine epidemic diarrhoea (PED^6^) and swine acute diarrhoea syndrome (SADS^7^); paramyxoviruses such as Hendra^8^ and Nipah^9^; and filoviruses like Marburg^10^ and Ebola^11^. Four families of viruses that are pathogenic for other mammalian species (*Coronaviridae*^12^, *Paramyxoviridae*^13^, *Rhabdoviridae*^14^ and *Filoviridae*^15^) may also have originated in bats. These viruses often cause serious disease in their secondary hosts, but most do not appear to cause clinical signs or pathology in bats^16–18^, suggesting that uniquely benign relationships have co-evolved between the viruses and their primary bat hosts^19,20^. While relatively little is known about the dynamics of viral infections in bats, these viruses may be maintained in bat populations as a result of either persistently infected individuals, reinfection after waning immunity, or spatial transmission dynamics^21,22^.

The rare spill-over of bat viruses to other animals may require a “perfect storm” of conditions that include increased contact between bats or fomites and other mammals, possibly due to human impacts on habitat quality^23^, and the ability of the virus to infect, replicate, and transmit in the secondary host. The rate of viral shedding and the amount of detectable virus associated with bat colonies fluctuates, with periodic increases often linked to parturition, waning maternal immunity, nutritional stress or increased energy consumption^17,24–29^. Increased shedding of virus by a colony of bats may reflect an increase in the proportion and number of susceptible individuals, or an increase in the replication of persistent or latent virus normally suppressed by the host. For herpesviruses, reactivation from latency is linked to perturbations caused by a variety of physiological, immunological and psychological stressors^30^. The mechanisms that trigger the reactivation of latent or persistently infecting viruses are not clearly understood, but the increased shedding of viruses is correlated with some incidents of spill-over of bat viruses to other animals^31^.

The Canadian prairies are home to three species of bats, including the little brown bat (*Myotis lucifugus*), big brown bat (*Eptesicus fuscus*), and northern long-eared bat *(Myotis septentrionalis*). All three species hibernate from October to May, sometimes in shared hibernacula. We recently demonstrated that ~30% of hibernating *M. lucifugus* sampled over two years from hibernacula in Manitoba were infected with a coronavirus (*Myl*-CoV), which persisted at low levels in the intestine^32^. A closely related coronavirus also infects *E. fuscus*^33^.

Whereas bats appear to be relatively resistant to viral infections, a cold-adapted fungus that was recently introduced to North America has caused widespread mortality in some species of bats in eastern United States and Canada^34–37^. The fungus (*Pseudogymnoascus destructans)* causes white-nose syndrome (WNS) in hibernating bats, which is characterized by the growth of white fungal mycelia on the face and exposed skin of the wings and tail membranes. The visual and microscopic effects of *P. destructans* on the skin of the wings are associated with increased expression of several genes devoted to innate immunity and inflammation in wing tissue^38,39^. Profound systemic effects include dehydration, hypovolemia, metabolic acidosis, and fat depletion, which can lead to death^40–42^. Other systemic effects of bat WNS include an accumulation of neutrophils in the lungs, which is accompanied by an increase in the expression of several cytokine genes^43^ suggesting that even the most severely afflicted hibernating bats are capable of at least some systemic immune response to fungal infection.

Previous studies on other species have demonstrated that a fungus and a virus could interact during co-infection and affect each other^44,45^. Similar interactive impacts of co-infection with *P. destructans* and viruses on bat immune responses are not known. We used *M. lucifugus* experimentally-infected with *P. destructans* and/or naturally infected with *Myl*-CoV as a model to understand how co-infections influence bat-virus interactions. This system allows us to avoid confounding factors of direct pathogen-pathogen interactions, because the fungus affects the skin, while the coronavirus infections occur internally, almost exclusively in the ileum and lungs^32^. We hypothesized that co-infection would alter the molecular response of bats to a persistent viral infection, and that viral shedding would change as a result of the increased or disrupted host immune response. To test this prediction, we examined tissues collected from *M. lucifugus* at the termination of an earlier study that quantified the effects and pathogenesis of *P. destructans* in hibernating bats experimentally infected with the fungus^37^, some of which were naturally infected with *Myl-*CoV^32^. This combination of uninfected, virus-infected, fungus-infected and co-infected *M. lucifugus* allowed us to test our hypothesis that host responses to co-infection are synergistic and not simply additive.

## Results

Quantitation of *Myl-*CoV and *M. lucifugus* RNA through reverse transcription quantitative PCR (RT-qPCR) and dual-RNA-sequencing indicated that co-infected bats had significantly higher levels of *Myl*-CoV RNA than bats infected with virus alone. The amount of *Myl-*CoV RNA correlated with the severity of WNS pathology in co-infected bats. This phenomenon was associated with specific molecular responses to co-infection, even in the intestines of bats where only one of the two pathogens was directly interacting with the host tissue. The levels of antibodies against *Myl*-CoV nucleocapsid (N) protein were also higher in co-infected bats. Each key result is discussed in detail below.

### Bats co-infected with the fungus *P. destructans* and the virus *Myl*-CoV contained higher levels of *Myl*-CoV RNA

**Figure 1:**
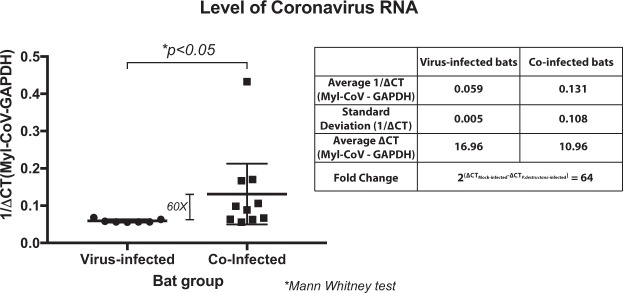
Effect of white-nose syndrome on level of *Myotis lucifugus* coronavirus (*Myl*-CoV) RNA in hibernating little brown bats (*M. lucifugus*). Relative transcript levels for the coronavirus RNA polymerase gene for each bat are depicted as reciprocal of Cycle threshold (Ct) normalized separately (ΔCt) for levels of glyceraldehyde 3-phosphate dehydrogenase (GAPDH) transcripts in each sample. The horizontal bar represents the mean while the vertical bar indicates standard deviation from the mean. Significance (*p* value) is as calculated with an independent Mann-Whitney test. Virus-infected bats had lower 1/ΔCt values for coronavirus RNA than co-infected bats. The average fold-differences between virus-infected and co-infected bats were calculated from the difference between the average ΔCt values.

*Myl*-CoV genomic RNA was detected in bats infected only with *Myl-*CoV (virus-infected; 7/18), co-infected bats (European *P. destructans* (3/13), or with North American *P. destructans* (7/16)^37^). There was no difference in the frequency of *Myl*-CoV detected among these treatments (*p-value* = 0.801). We pooled bats infected with the two *P. destructans* isolates for all further analyses and tested whether co-infection with *P. destructans* and *Myl-*CoV correlated with an increase in viral replication. Our RT-qPCR data showed that the co-infected bats contained 60-fold more *Myl-*CoV RNA on average than the virus-infected bats (Mann Whitney test; *p-value* = *0.014*; Fig. 1).

Relative quantities of *Myl*-CoV RNA detected in the ileum of the virus-infected bats were low and showed low variation (Standard Deviation of 1/ΔCT = 0.005), compared to the relative quantities of *Myl*-CoV RNA in the co-infected bats (Standard Deviation of 1/ΔCT = 0.108; Fig. 1). The severity of WNS fungal pathology varied in co-infected bats, and we therefore tested whether relative quantities of viral RNA in the ileum correlated with the severity of WNS symptoms. Levels of WNS severity were scored based on fungal hyphae on the wings, secondary bacteria in wing lesions, oedema, necrosis and inflammation in wing lesions, and levels of neutrophils in lung, spleen and liver interstitium. Severity scores for wing tissue, secondary bacteria in lesions, and neutrophils in the lung interstitium positively correlated with relative amounts of coronavirus RNA in hibernating bats (Table 1).

**Table 1:** Correlation between level of *Myotis lucifugus* coronavirus RNA and disease severity of white-nose syndrome (WNS) in co-infected *M. lucifugus*, based on three measures of WNS severity and pathology.

Correlate	Level of coronavirus RNA		
	Pearson Correlation^a^	Significance	N
Virus-infected/Co-infected	−0.610	**0.009**	17
Average hyphae score	−0.630	**0.016**	14
Average bacterial score	−0.680	**0.007**	14
Lung interstitial neutrophils	−0.618	**0.043**	11

^a^Pearson’s coefficients were calculated for the ∆Ct levels for cytokine transcripts for bats in each treatment class and lung interstitial neutrophil scores and mean bacterial and hyphae scores for 5 wing sections for each bat.

### Bat responses to co-infection exceed the sum of responses to virus or fungal infection alone

**Figure 2:**
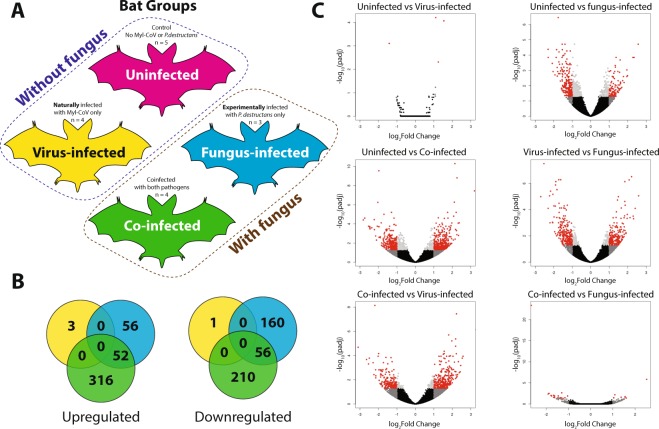
Co-infection of little brown bats (*Myotis lucifugus*) with *M. lucifugus* coronavirus (*Myl-*CoV) and *Pseudogymnoascus destructans* results in non-additive patterns of gene expression compared to sole infection with the virus or fungus. (**A**) Experimental design, showing the four treatments of little brown bat (*Myotis lucifugus*) established by experimental inoculation with *Pseudogymnoascus destructans* and by qPCR detection of persistent *Myl*-CoV infections: uninfected, virus-infected, fungus-infected and co-infected. (**B**) Differential gene expression identified by DESeq2 among virus-infected, fungus-infected and Co-infected bats as compared to the change each exhibited relative to uninfected bats. (**C**) Differential gene expression among the four treatments, detected by DESEQ2 and visualized in volcano plots. The log of the adjusted *p*-value is plotted as a function of the log ratio of differential expression. Colored data points represent different groups of genes based on fold change and false discovery rate (FDR) cutoff; red (>2 fold change, FDR <0.05), dark grey (>2 fold change, FDR > 0.05), light grey (<2 fold change, FDR < 0.05), black (<2 fold change, FDR > 0.05).

To determine the extent to which *Myl*-CoV and *P. destructans* infection interact to influence gene expression in bat intestines, we performed a transcriptomic analysis on bat intestines comparing gene expression among the uninfected, virus-infected, fungus-infected, and co-infected treatments (Fig. 2(A)). RNA sequencing resulted in ~700 million paired-end reads passing filters, 65% of which aligned to the *M. lucifugus* genome (Table S1). Pairwise differential gene expression varied widely among the four treatments with relatively low overlap in differentially expressed transcripts (Fig. 2B,C), Supplementary Fig. 1). Similar transcript expression occurred between the uninfected and virus-infected bats, and between the fungus-infected and co-infected bats.

The fungus-infected bats exhibited a much stronger response, differentially expressing 324 transcripts compared to the uninfected bats (Table S3). These transcripts were enriched for only two gene ontology (GO) terms (cell-cell junction and plasma membrane part; Table S8). The co-infected bats differentially expressed 634 transcripts relative to the uninfected bats (Table S4). These transcripts showed significant enrichment for 16 GO terms (Table S8). The co-infected and fungus-infected bats shared 108 similar differentially expressed transcripts and overlapped in one enriched GO term relative to the uninfected bats (plasma membrane part; Table S8).

### Effect of infection with the fungus *P. destructans* on the expression of genes linked to innate responses in the intestines of bats infected with the virus Myl-CoV

When we directly compared responses of bats among the four treatments, response of the virus-infected bats differed strongly from the responses of fungus-infected or co-infected bats (virus-infected vs. fungus-infected: 461 differentially expressed transcripts and 9 significantly enriched GO terms; virus-infected vs. co-infected: 473 transcripts and 43 enriched GO terms; Tables S5, S6, S7; Supplementary Fig. 1). These differences in gene expression patterns included genes that clustered in two processes relevant to host-pathogen interactions – the mitogen-activated protein kinase (MAPK) pathways and cytokine and innate immune responses. Table 2 lists genes from the two processes that were significantly either up or down-regulated when virus-infected bats were compared to co-infected bats. For the MAPK pathway-related transcripts, genes such as RSU1 and RERG were up-regulated while those, such as STYK1, RRAD, MAP3K and SRC were down-regulated. For cytokine-related genes several transcripts were suppressed. When we compared the expression of the same genes for bats with WNS (combining fungus-infected and co-infected bats) and all bats without WNS (combining uninfected and virus-infected bats), we found similar differences (last two columns of Table 2). This suggested that superficial infection with fungus, *P. destructans*, was the driving factor for altered gene expression in the bat intestines.

**Table 2:** RNA-sequencing identified differential expression of transcripts related to the MAPK pathway and to cytokine-related processes, comparing gene expression in the ileum of little brown bats (*Myotis lucifugus*) infected only with the *M. lucifugus* coronavirus (*Myl-*CoV; virus-infected) or co-infected with *Myl-*CoV and *Pseudogymnoascus destructans*. The last 2 columns show the same comparisons made after grouping bats that were not exposed to the fungus, and bats that were exposed to the fungus and exhibiting symptoms of WNS (irrespective of their viral infection status).

Ensembl Gene Name	Ensembl Description	Virus-infected vs. Co-infected		All bats “without fungus” vs. all bats “with fungus”^a^	
		Log_2_ Fold Change^b^	Padj	Log_2_ Fold Change^b^	Padj
*MAPK pathway-related transcripts*					
STYK1	serine/threonine/tyrosine kinase 1	−1.268	0.025	−1.516	<0.0001
RSU1	Ras suppressor protein 1	1.102	0.004		
RRAD	RRAD, Ras related glycolysis inhibitor and calcium channel regulator	−1.297	0.028	−1.3	0.025
RERG	RAS like estrogen regulated growth inhibitor	1.562	0.018	1.416	0.005
MAP3K11	mitogen-activated protein kinase 11	−1.14	0.040	−1.13	0.0002
SRC	SRC proto-oncogene, non-receptor tyrosine kinase	−1.539	0.013	−1.297	0.0037
*Cytokine-related transcripts*					
IRF1	Interferon regulatory factor 1	−1.551	0.001	−1.444	0.0001
IFI6	Interferon alpha inducible protein 6	−1.798	0.014	−1.352	0.039
IL22RA1	Interleukin 22 receptor subunit alpha 1	−1.411	0.015	−1.314	0.002
SOCS6	Suppressor of cytokine signaling 6	−1.278	0.008	−1.534	<0.0001

^a^(Uninfected + virus-infected) vs. (fungus-infected + co-infected)

^b^Positive log2 fold-change values indicate higher expression in the second listed treatments relative to the first.

**Figure 3:**
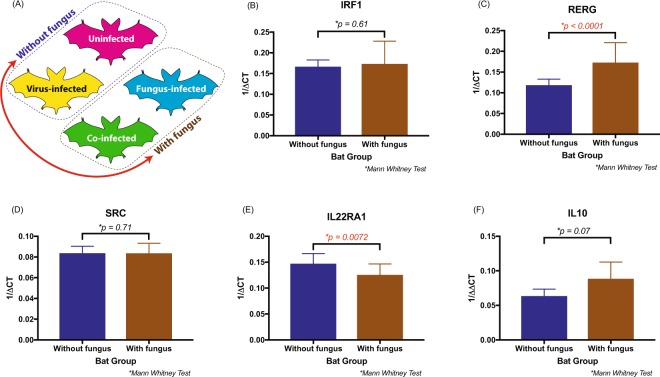
Effect of white-nose syndrome (WNS) on the levels of immune genes IRF1, RERG, SRC, IL22RA1 and IL10 expressed in the ileum of little brown bats (*Myotis lucifugus*). (**A**) Summary of the four treatments, with a red arrow indicating the two groups (“with fungus” and “without fungus”) that were compared. (**B**–**F**) The relative transcript levels of each gene for bats with and without WNS, depicted as reciprocal of Cycle threshold (Ct) normalized separately (ΔCt) for levels of transcripts for GAPDH in each sample. Statistical significance was calculated based on the independent Mann Whitney test. The difference in the two groups was significant for RERG and IL22RA1 genes.

To confirm the results of the RNA-seq analysis, we selected 4 genes from Table 2, namely IRF1, RERG, SRC and IL22RA1, to be verified by RT-qPCR. We also included interleukin 10 (IL10) due to its biological relevance to immune regulation and because we had previously observed an increase in its expression related to fungal infection^43^. As we wanted to confirm whether WNS was driving gene expression in the intestines of bats, we performed a two-group analysis for the RT-qPCR data. We combined all the bats without WNS into a single group (uninfected + virus-infected) and all the bats with WNS into the other group (fungus-infected + co-infected; Fig. 3(A)). Expression of Ras-like estrogen regulated growth inhibitor (RERG) increased while expression of Interleukin 22 receptor subunit alpha 1 (IL22 RA1) genes decreased in bats with WNS, irrespective of viral infection (Fig. 3C,E). Expression of the immune modulatory cytokine IL10 tended to be higher in bats with WNS than in bats without WNS, but the difference was not statistically significant (*p*-value = 0.07) (Fig. 3(F)).

### White-nose syndrome is associated with increased coronavirus antibody levels in the co-infected bats

**Figure 4:**
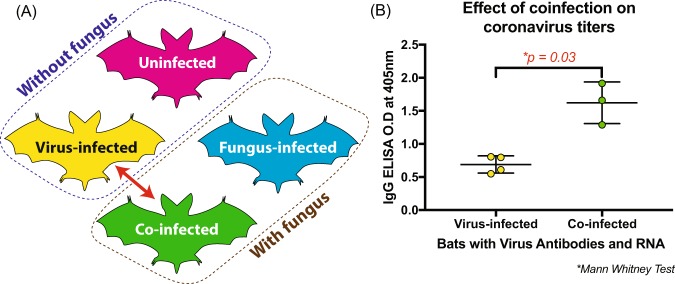
Little brown bats (*Myotis lucifugus)* coinfected with *M. lucifugus* coronavirus (*Myl*-CoV) and *Pseudogymnoascus destructans* produce more antibodies against *Myl*-CoV than bats infected only with *Myl-*CoV. (**A**) Diagram summarizes the four treatments; the red arrow shows the two groups between which antibody levels were compared. (**B**) Antibody levels against the *Myl*-CoV N protein detected by antibody capture ELISA expressed as optical density (O.D.) values at 405 nm. Co-infected bats had significantly higher antibody levels than bats infected only with *Myl*-CoV (independent Mann Whitney test; *p value* = 0.03).

In 2017, we performed a similar study, experimentally exposing 63 *M. lucifugus* to *P. destructans* as described in Warnecke *et al*.^37^. We performed IgG ELISA on blood plasma to detect *Myl*-CoV (coronavirus) N protein antibodies and found that 21/63 were positive for antibodies against the coronavirus. Of those 21 bats, 7 had detectable coronavirus RNA in their intestines suggesting an active infection, and 3 out of the 7 had been experimentally infected with *P. destructans* during the course of the study. We compared the ELISA optical density (O.D.) values of these virus-infected bats to co-infected bats (Fig. 4(A)) and found that the presence of *P. destructans* was associated with increased levels of coronavirus antibodies (Mann Whitney test, *p value* = 0.03; Fig. 4(B)).

## Discussion

Our findings suggest that systemic responses of bats to WNS results in increased coronavirus replication and consequently, increased viral shedding, which may lead to subsequent infection of susceptible animals. Coronavirus infection may in turn increase the severity of WNS pathology. This is the first study to examine the systemic effects of co-infection on either bat coronavirus or WNS, and our results raise important questions in regard to zoonotic spillover events. Although events of successful viral spillover to distantly related species are thought to be extremely rare, in recent years several coronaviruses have spilled over, including SARS-CoV^1^, MERS-CoV^2–5^, PEDV-CoV^6^ and SADS-CoV^7^. These viruses are thought to have originated in bats. In addition, circumstantial evidence suggests that most alpha and beta coronaviruses that parasitize other mammals may have originated in bats as well^46^. If so, then understanding host-pathogen interactions between bats and coronaviruses could inform our ability to predict or manage the risk of spillover. In this study, we showed that a coronavirus exhibits low activity in its natural host, *M. lucifugus*, but that co-infection with a fungus increases the quantity of viral RNA in the intestines. We have no reason to expect zoonotic transmission of the coronavirus i.e. *Myl*-CoV, but similar co-infection mechanisms may operate in tropical bat species harbouring potentially zoonotic viruses.

**Figure 5:**
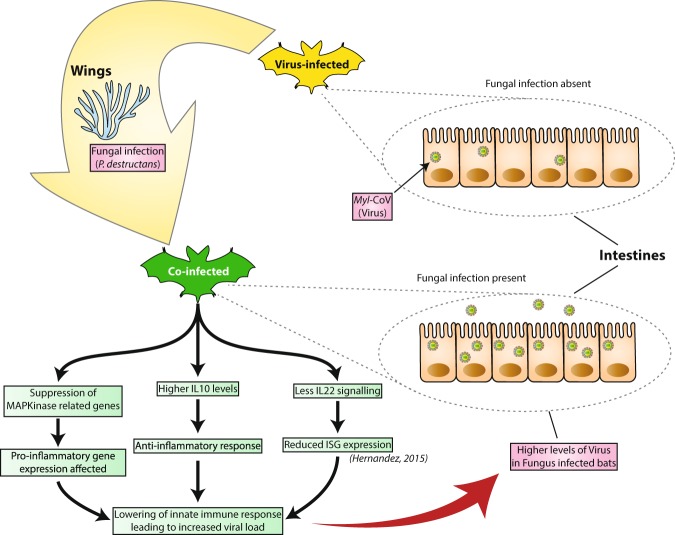
Hypothesized model of pathways involved in increased coronavirus shedding and white-nose syndrome (WNS) severity in little brown bats (*Myotis lucifugus*) co-infected with *M. lucifugus* coronavirus (*Myl-*CoV) and *Pseudogymnoascus destructans*. Diagram summarizes the changes observed by comparing co-infected bats with virus-infected bats. Bats with persistent *Myl*-CoV infection exhibit relatively low viral shedding. When bats are also infected with *P. destructans* (shown in yellow arrow) and develop WNS, the level of coronavirus increases. There is a change in the level of some immune genes, such as IL22, RERG and possibly IL10, which may have an effect on immune response and cell proliferation. The increase in coronavirus levels in co-infected bats is possibly due to the bats’ systemic response to WNS reducing innate anti-viral responses.

Our results suggest that secondary skin infection with the fungus, *P. destructans*, substantially increases the level of viral RNA in the intestine of hibernating bats. We showed that infection of the skin with *P*. *destructans* can cause profound changes in gene expression in the intestines, despite a lack of direct contact between intestinal tissue and the fungus. Infection with *P. destructans* causes modulation of a number of immune responses, including down-regulation of interleukin and cell proliferation genes which may compromise bats’ ability to suppress viral activity (Fig. 5). Taken together, our results have implications for epidemiological studies of *P. destructans*, the WNS fungus and for research into viral spillovers, which should consider the potential implications of co-infections that increase viral shedding.

Complex strategies allow viruses to remain endemic in populations. These include a continuously replenished source of susceptible hosts for viruses that cause short-term acute infections with long-lasting immunity (e.g. measles virus), antigenic drift of virus (e.g. influenza virus) or waning immunity (e.g. respiratory syncytial virus) that allows reinfection, and long-lasting latent (e.g. herpesviruses) or persistent infections (e.g. pestiviruses) with sustained or periodic shedding. It is not yet clear how bat viruses are maintained in their natural host populations, or how they avoid extinction as host populations become immune and less susceptible. Persistent infections can be established in cultured cells with viruses that may have originated in bats, including Ebola virus^47^ and SARS-CoV^48–50^, but whether these viruses persist in their primary hosts is not known. Studies of persistence of bat viruses in infected bats have produced equivocal results. The lack of direct evidence supporting specific models of persistence or transmission dynamics represents a major knowledge gap in bat-virus ecology^21^.

We maintained *M. lucifugus* in controlled laboratory hibernation chambers for four months during these experiments, and we detected the coronavirus i.e. *Myl*-CoV, at the end of hibernation. These data imply that the coronavirus can persist in its host for at least the duration of hibernation, particularly as nucleotide variability among the detected coronavirus isolates showed that spread of coronavirus among bats within a chamber was unlikely^32^. In an extensive study of New World alphacoronaviruses, no target viruses were detected in the rectal swabs of individual bats sampled over time^46^, suggesting that persistence and intensity of shedding varies among species or viruses. The authors concluded that the targeted coronaviruses do not persist in their hosts but are maintained in populations by the introduction of new susceptible individuals. However, their results could also reflect viral persistence in individual animals, with low baseline levels of virus replication and undetectable shedding interspersed with periods of increased replication and shedding that did not occur during the sampling period.

Periodic or seasonal increases in virus shedding associated with parturition, lactation, nutritional deprivation or environmental stress^21,29^ suggest persistent or latent viruses may be activated by hormonal or other systemic cues. Direct evidence linking a specific trigger to increased shedding has not yet been found. However, viral replication in rodent and bat cells persistently infected with Ebola virus increased greatly following modulation of the Ras/MAPK pathway with lipopolysaccharides or phorbol esters, and with the resulting suppression of the cells’ interferon response^47–49^. In experimental systems, the immune modulatory cytokine IL10 also influences viral persistence and replication^51–53^, although more study is required to clarify the effects of circulating cytokines on the replication of persistently infecting viruses. Nevertheless, these results suggest that circumstances which induce anti-inflammatory cytokines or suppress anti-viral innate responses, may provide a trigger for increased shedding of persistently infecting virus.

We discovered that bats with WNS (fungus-infected and co-infected) had significantly lower intestinal levels of transcripts for IL22RA1 and other interferon-related genes as compared to uninfected bats, and we observed the same trend in IL10 (although it was not significant; *p value* = 0.07). IL22RA1 is the receptor present on host cells, including intestinal cells, which help in initiating cellular signalling in response to IL22 produced by T-cells^54^. IL-22 leads to an increase in anti-microbial peptide production, cellular protection against damage and increases cellular proliferation^55^. Therefore, reduced IL-22 signalling in the intestines of bats with WNS, might suppress the bat defences that control the coronavirus infection. Additionally, previous studies have shown that the anti-inflammatory gene, IL10, is expressed more in the lungs of bats with WNS than in bats without it^43^. We saw a similar trend with the levels of IL10 in the intestines which might play a role in suppressing the immune response against the coronavirus. Another altered cytokine gene which was of interest was the suppressor of cytokine signalling-6 (SOCS6) gene. Fungal-infected bats showed lower levels of SOCS6 transcripts, lack of which has been implicated in mild growth retardation in mice^56^. Overall, our results suggest that WNS triggers changes in gene expression in the ileum (Fig. 5). These may influence expression of interferon-stimulated-genes (ISGs), thereby leading to increased viral replication at the site of viral persistence. Interferon-related transcripts were more highly expressed in the ileum of virus-infected bats that did not have WNS, suggesting that the bat’s response to WNS causes down-regulation of interferon activity. Interferons may control coronavirus replication, as seen in cases of SARS-CoV^57^ and MERS-CoV^58^. Therefore, a decrease in interferon activity might cause an increase in coronavirus (*Myl*-CoV) replication. In addition to interferon-related genes, we also found that RERG, which is related to growth inhibition, was upregulated in the fungus-infected bats when compared to virus-infected bats. Upregulation of RERG could affect the rate of cell proliferation in the intestines^59^. Finally, this cascade of responses is associated with increased severity of WNS symptoms.

Bats with WNS experience a range of systemic disturbances including dehydration, hypovolemia, metabolic acidosis and fat depletion^40,41^, neutrophil infiltration of the lung interstitium, and increased expression of transcripts related to anti-microbial and pro- and anti-inflammatory cytokines^43^. Taken together, this evidence suggests that hibernating bats respond systemically to superficial fungal infection, and this hypothesis is further supported by our observations of altered gene expression in the ileum of fungus-infected bats.

Based on our results, we propose a model for how secondary infections may increase the replication and subsequent shedding of persistently infecting virus (Fig. 5). The establishment of WNS (or other secondary infection) impacts the tissue with which that pathogen interacts (in the case of *P. destructans*, the skin). Direct interactions between the host and the secondary pathogen are limited to the affected tissue, but the systemic response to the disease triggers a cascade of immune responses, including increased release of cytokines or neutrophils. Affected cells such as intra-alveolar macrophages in the lungs or cells lining the intestine, may produce pro- or anti-inflammatory molecules and influence cells that harbour viral genomes. This cascade of host responses disrupts the equilibrium between the persistently infecting virus and the cell’s innate immune response, leading to a dramatic increase in the expression of coronavirus (*Myl-*CoV) replication.

Our assays were unfortunately limited to analysing viral and cytokine transcripts rather than protein, because reagents for detecting bat viral and host proteins are not yet available. We were not able to perform serial dilutions of the plasma to precisely quantify anti-viral titre due to the limitation in the amount of plasma obtained from each bat. The sample size was also small for this assay, because only 7 of the sampled bats had detectable levels of coronavirus in their intestines and were positive for viral antibodies. Despite these limitations, we demonstrated higher antibodies against the coronavirus in the plasma of co-infected bats when compared with virus-infected bats. This increased antibody level in co-infected bats might reflect an adaptive immune response to increased coronavirus replication in the intestines.

Our proposed hypothesis for the mechanism driving increased viral replication following pathogenic co-infection was worth testing, but our results are also consistent with an alternative hypothesis. Increased viral replication or viral load may affect the severity and population-level impacts of WNS. Bat mortality following the arrival of WNS varies widely from site to site, with populations decreasing from 30% to 99%^35^. Variation in the microclimate, and other ecological factors may drive some of this variation^60^, but our data suggest that cryptic viral infections may also play a role in determining survival rates for bats hibernating in sites colonized by *P. destructans*. We recommend that future studies on population-wide impact of WNS incorporate viral sampling to help better understand the role of co-infections on bat populations in the wild.

## Materials and Methods

### Sample acquisition

Fifty-four male *M. lucifugus* were collected from a WNS-free cave in Manitoba, Canada in November 2010. Details of the experimental design as well as protocols for collecting and transporting bats, infection with *P. destructans*, maintenance of bats in hibernation and sample collection have been described previously^37,43^. Briefly, bats in groups of 18 were either sham-inoculated or inoculated with North American or European isolates of *P. destructans*. Bats were housed at 7 °C and >97% relative humidity with *ad libitum* water. All bats were equipped with data loggers to monitor skin temperatures. Bats were euthanized during the experiment when humanely required or at the termination of the experiment 120 days after inoculation. Immediately following euthanasia samples from segments of wing as well as various tissues were preserved in RNAlater (Qiagen, 76016) or in formalin. Samples in RNAlater were kept at −20 °C until they were processed. North American and European isolates of *P. destructans* caused similar disease outcomes^37^, so we did not differentiate between the strains in subsequent analysis. The procedures for care, handling and euthanasia of bats were approved by the University Committee on Animal Care and Supply of the University of Saskatchewan (Protocol #20100120). Bats were collected under the province of Manitoba Wildlife Scientific Permit WB11145.

In 2017, a further 129 *M. lucifugus* were collected from a WNS-free cave in Manitoba, Canada in January under the Manitoba Sustainable Development Wildlife Scientific Permit No. SAR16009. Bats were euthanized during the experiment when humanely required or at the termination of the experiment 70 days after infection and a similar experiment was performed at the University of Winnipeg as described above (Protocol #AE08399).

### Histological classification

During necropsy, we collected representative samples for histopathology from all major organ systems. In addition, representative samples were taken from all areas of the wing and rolled on dental wax before placing in 10% neutral buffered formalin. Tissues were processed routinely for histology. Five µm sections were cut and stained with periodic acid-Schiff stain to highlight fungal hyphae. Liver and other tissues were processed routinely and stained with hematoxylin and eosin. Wings were scored on a scale of 0 to 5 with 5 being very severe with >50% of wing covered in fungal hyphae. We used a bacterial score from 0 to 5, with 5 indicating wide-spread and abundant bacteria being present in many areas within the dermis and underlying connective tissues. Average scores from 5 sections of wing were used for analysis. Interstitial lung neutrophil assessment was similarly evaluated on a scale of 0 to 5, with 5 being very severe. Average scores from the 5 sections were used for analysis.

### RNA Extraction

Tissues preserved in RNAlater were homogenized in 2 ml sealed vials with a 5 mm stainless steel bead, 0.1 g of 0.1 mm zirconia/silica beads and 350 μl Buffer RLT Plus (with β-mercaptoethanol, RNeasy Plus Mini Kit, Qiagen, 74136) using a Retsch MM400 Oscillating Mill at 30 Hz for 4 min. Total RNA was extracted following the manufacturers protocol. RNA integrity was assessed using RNA 6000 Nano Kit (Agilent, 5067-1511) with the Agilent 2100 Bioanalyzer.

### cDNA Synthesis

cDNA was synthesized from 1 μg of RNA (or less if concentrations were too low) per reaction using QuantiTect Reverse Transcription Kit (Qiagen 205313). cDNA samples were stored at −80 °C until they were used for PCR.

### Polymerase Chain Reaction (PCR)

Tissue samples were identified by their submission numbers with no reference to treatment class prior to analysis with PCR, so that evaluation of the results could not be inadvertently biased by knowledge of the treatment. We used semi-nested PCR to detect *Myl-*CoV. Primers were designed from the partial sequence of Rocky Mountain bat coronavirus replicase (accession number EF544563) (Table S9). The primary reaction used primers MyCVF1 and MyCVR1 to yield a 441 bp product. The secondary or nested reaction used primers MyCVF2 and MyCV R1 to give a 273 bp product. PCR were performed in a MJ Research PTC-200 thermal cycler using TopTaq DNA Polymerase (Qiagen, 200205). Each reaction (50 μl) contained 2 μl cDNA (or 1 µl primary reaction), 200 nM of each primer, 200 µM of each dNTP (Invitrogen, 10297018), TopTaq PCR buffer and 0.25 μl TopTaq. The thermal profile for the primary reaction was: 94 °C for 3 min (denaturation), followed by 30 cycles of 94 °C for 30 sec, 45 °C for 30 sec (annealing), 72 °C for 1 min and finally 72 °C for 10 min. The thermal profile used for the secondary reaction was 94 °C for 3 min (denaturation), then 30 cycles of 94 °C for 30 sec, 55 °C for 30 sec (annealing), 72 °C for 1 min and finally 72 °C for 10 min. PCR products were analyzed on ethidium bromide stained 1.0% agarose gels (Invitrogen 15510-027 in 0.5X TBE). PCR products were purified using MinElute PCR Purification Kit (Qiagen, 28006) and verified by sequencing (Macrogen, Korea). If more than one DNA band was present, the appropriate size band was cut out and purified using QIAquick Gel Extraction Kit (Qiagen, 28706) before sequencing.

### Reverse-Transcription Quantitative PCR (RT-qPCR)

The Stratagene MX3005P qPCR System was used in conjunction with QuantiFast SYBR Green PCR Kit (Qiagen 204056). We quantified coronavirus with RNA primers MyCVF2 and MyCV R1 (Table S9). For initial experiments data were normalized to two transcripts – glyceraldehyde-3-phosphate dehydrogenase (GAPDH) and beta-actin^43^. As there were no differences in results, all subsequent experiments used only GAPDH as a normalizer using primers GAPDH US and GAPDH DS (designed for use in humans but also amplify *M. lucifugus* transcripts – Table S9). As well, a no-template (negative) control was included with every set of primers. Each 25 μl reaction contained: 1 μM of each primer set, 12.5 μl SYBR Green Master Mix and 8.5 μl of diluted cDNA.

To verify the RNAseq data, cDNA from ileum samples in which coronavirus RNA had been detected via RT-qPCR were analysed using the following primers, IL22RA1, IRF1, RERG and SRC (for sequence of primers see Table S9). Primers were designed by aligning primers described for quantitating human cytokines (PrimerBank) with annotated transcripts of *M. lucifugus* genes: c-jun (Accession number: XM_006096110.1), cyclin D1 (XM_006098046.1), IL10 (XM_006094865.1) and TNF alpha (XM_006104644.1). The interferon beta primers were designed using the annotated transcript for the *E. fuscus* gene (XM_008145044.1), which also amplify transcripts from *M. lucifugus*. Primer efficiencies were determined from cycle threshold (Ct) values of purified PCR products serially diluted and re-amplified. Primers amplified targets with an efficiency of about 100% and in all cases the identities of the PCR products were confirmed by their specific dissociation temperature, specific sizes on agarose gels and by sequencing.

We observed primer-dimers in some reactions in addition to the PCR product. The dimers dissociated at 77 °C, while the specific coronavirus polymerase product dissociated at 83 °C. To avoid false positives due to primer-dimers, the thermocycler was programmed to read at 80 °C (in the cycle after the primer-dimer had dissociated, and before dissociation of the target product). The thermal profile used was 95 °C for 5 min followed by 40 cycles of 95 °C for 10 sec, 60 °C for 30 sec (readings taken at 80 °C), and a final cycle of dissociation of product 95 °C for 1 min, 55 °C for 30 sec and 95 °C for 30 sec (readings taken at every degree between 55 °C and 95 °C). Only results from reactions that yielded unambiguous results were used for analysis.

### RNA-seq Analysis

To explore the mechanisms driving high virus load in bats with WNS, we performed RNA-seq analysis which could potentially screen all targets in the bat intestinal cells. We targeted the ileum transcriptome because this is the tissue in which *Myl*-CoV is present in detectable concentrations^32^. Extraction of RNA from ileum tissue, which includes the ileum and potential gut contents have been described in previous sections. Bats were screened for *Myl*-CoV using RT-qPCR, and bats were assigned post hoc to treatment groups representing four infection histories (Fig. 1(A)): 1) Uninfected (bats were not infected with virus or fungus; n = 5), 2) Virus-infected (bats were naïve to the fungus but had a persistent *Myl*-CoV infection; n = 4), 3) Fungus-infected (bats were experimentally infected with *P. destructans* and no virus was detected*;* n = 3), or 4) Co-infected (bats with persistent *Myl*-CoV infections that were also experimentally infected with *P. destructans*; n = 4). All samples had adequate RNA quality for sequencing (i.e. RIN value >7).

### RNA isolation

Tissues were homogenized in 2 ml sealed vials with a 5 mm steel bead, 0.1 g of 0.1 mm zirconium silica beads, 350 µL of RLT buffer (with β-mercaptoethanol) (RNeasy Plus Kit, Qiagen) using a Retsch MM400 tissue homogenizer at 30 Hz twice for 2 minutes each. Total RNA from tissues was extracted using the procedure provided with the RNeasy Plus Kit.

### cDNA library preparation and RNA-sequencing

Total RNA was sent to The Centre for Applied Genomics at The Hospital for Sick Children (Toronto, Canada). RNA quality was assessed using a Bioanalyzer (Agilent Technologies). We retained all samples with a DV200 (percentage of RNA fragments greater than 200 nt) greater than 85% (Table S1), discarding one Co-infected sample with a DV200 = 42%. Poly(A) mRNA was enriched using oligo dT-beads, and cDNA libraries were prepared using the NEBNext Ultra Directional RNA Library Prep Kit for Illumina (New England BioLabs). Barcoded libraries were pooled in equimolar quantities, and the sixteen libraries were sequenced on three lanes of a HiSeq. 2500 System (Illumina Inc.), which generated 126 bp paired-end reads.

### RNA-sequencing read alignment and analysis

We used FastQC v0.11.5^61^ to assess sequence quality and Trimmomatic v0.36^62^ to remove the adapter sequences and low-quality bases from reads with the following settings: Illumina clop:2:30:10, leading:3, tailing:3, slidingwindow:4:15, minlength:36. We used TopHat v2.1.1^63^ to align the trimmed paired-end reads from each library, separately, to the Ensembl *M. lucifugus* genome sequence (Myoluc2.0^64^) in strand-specific mode (fr-firststrand) with mate-inner-dist values specific for the insert size of each library. We used featureCounts^65^ to count reads mapped to the Myoluc2.0 genome annotation in strand-specific mode (reversely stranded), counting paired-end reads as fragments, counting only those fragments where both reads aligned successfully, counting multi-mapping fragments, and excluding chimeric fragments. We assessed the variability within and between the treatments using the R package SARTools v.1.3.0^66^. The featureCount-estimated gene counts were transformed by a variance stabilizing method (VST) using SARTools.

We identified differentially expressed genes between each of the treatments using DESeq2 v.1.12.3, run in SARTools. Custom SARTools-based DESeq settings included: cooksCutoff = TRUE (perform outliers detection), independentFiltering = TRUE, alpha = 0.05 (threshold of statistical significance), pAdjustMethod = BH (benjamini hochberg *p*-value adjustment method), and locfunc = median (estimate size factors). Differentially expressed genes were identified as having a fold-change >2 and false discovery rate (FDR)-corrected *p*-values < 0.05^67^. We produced volcano plots representing the differential expression comparisons by plotting the log of the adjusted p value as a function of the log ratio of differential expression. We used the Ensembl gene IDs identified by DESeq2 as input for the web-based g:Profiler^68^ to test for gene ontology (GO) term enrichment among the differentially expressed genes, using a FDR significance threshold <0.05. These GO-terms and their corresponding *p*-values were used in REViGO^69^ to visualize significant enrichment of biological processes.

### IgG capture ELISA against *Myl*-CoV N protein

Purified, glutathione-s-transferase (GST)-tagged *Myl*-CoV N protein expressed in infected *E. coli* BL21 cells was used as positive antigen, and GST-tagged protein expressed in uninfected BL21 cells was used as negative antigen. 96-well Costar high-binding round-bottom assay plates were coated with 0.05 µg/well of either antigen diluted in 0.1 M phosphate buffered saline (pH 7.4) in a total volume of 100 µl. Plates were covered and incubated overnight at 4 °C and washed three times with 300 µl of PBS-Tween 20 (0.1%) immediately prior to use. Serum samples were diluted to 1:100 in PBS-Tween 20 (0.2%) supplemented with 5% fetal bovine serum (Gibco, Thermofisher). 100 µl of each sample was added in parallel to a positive and negative antigen plate and incubated at 37 °C for one hour and washed as above. A peroxidase-labelled goat anti-bat IgG secondary antibody (0.05 µg in 100 µl per well, Bethyl labs) was added, incubated for one hour at 37 °C and washed as above. Peroxidase substrate (2,2’-azino-bis (3-ethylbenzthiazoline-6-sulfonic acid)) was added to each well and colour development was quantified 30 minutes later by measuring the optical density at 405 nm using an ELISA microplate reader. The ELISA cut-off value (0.39) was calculated as the [(mean bat plasma O.D. values for bats that were PCR-negative for *Myl*-CoV in the ileum) + (3x standard deviations of those O.D values)].

### Statistical analysis

Data from RT-qPCR and histopathological scores were analysed with SPSS Statistics version 23. The relative levels of a transcript for each bat were calculated as RT-qPCR Cycle threshold (Ct) normalized separately (ΔCt) to the “house-keeping” gene GAPDH. A ΔCt reduction of one (1) indicates an approximately two-fold higher concentration of RNA. The significance of differences of mean values of ΔCt between co-infected bats and virus-infected bats were determined using an independent-samples Mann-Whitney U test. We calculated Pearson’s coefficients to test the correlation between ΔCt levels for coronavirus polymerase cDNA for bats in each treatment class, and average scores for fungal hyphae, secondary bacteria, oedema, necrosis and inflammation in wing lesions, as well as bacteremia and levels of neutrophils in lung, spleen and liver interstitium.

### Ethical Statement

Bat studies were carried out in strict compliance with Canadian Council on Animal Care guidelines and the procedure for care, handling, and euthanasia of bats were approved by the University Committee on Animal Care and Supply of the University of Saskatchewan (protocol s#20100120).

## Electronic supplementary material

Supplementary Fig 1

Datasets S1-S9

## Data Availability

All RNA-seq fastq files have been submitted to the NCBI Sequence Read Archive database (accession number SRX3752319- SRX3752333). These data files will be released to the public upon acceptance of this manuscript for publication.
