# Supplementary material for: White-nose syndrome is associated with increased replication of a naturally persisting coronaviruses in bats
Source: Sci Rep. 2018 Oct 19;8:15508. doi: 10.1038/s41598-018-33975-x (PMC6195612; doi:10.1038/s41598-018-33975-x)
Supplement: Supplementary file 1 — Supplementary Fig 1 [file 41598_2018_33975_MOESM1_ESM.pdf]

Supplementary information

White-nose syndrome is associated with increased replication of a naturally persisting coronaviruses in bats

Christina M. Davy[1,2,Ψ], Michael E. Donaldson[1Ψ], Sonu Subudhi[3Ψ], Noreen Rapin[3Ψ], Lisa Warnecke[4,#a], James M. Turner[4,#b], Trent K. Bollinger[5], Christopher J. Kyle[6], Nicole Dorville[4], Emma Kunkel[4], Kaleigh Norquay[4], Yvonne Dzal[4], Craig K.R. Willis[4], and Vikram Misra[3]*

[1]Environmental and Life Sciences Graduate Program, Trent University, Peterborough, ON, Canada

[2]Ontario Ministry of Natural Resources and Forestry, Wildlife Research and Monitoring Section, Trent University, Peterborough, ON, Canada

[3]Department of Microbiology, Western College of Veterinary Medicine, University of Saskatchewan, Saskatoon, Saskatchewan, Canada

[4]Department of Biology, University of Winnipeg, Manitoba, Canada

[#a]Present address: Department of Animal Ecology and Conservation, University Hamburg, Hamburg, Hamburg, Germany

[#b]Present address: Institute for Land Water and Society, Charles Sturt University, Albury, New South Wales, Australia

[5]Canadian Wildlife Health Cooperative and Department of Pathology, Western College of Veterinary Medicine, University of Saskatchewan, Saskatoon, Saskatchewan, Canada

[6]Forensic Science Department, Trent University, Peterborough, ON, Canada

Ψ Contributed equally to the study

*Corresponding author, e-mail: vikram.misra@usask.ca

Supplementary figure 1

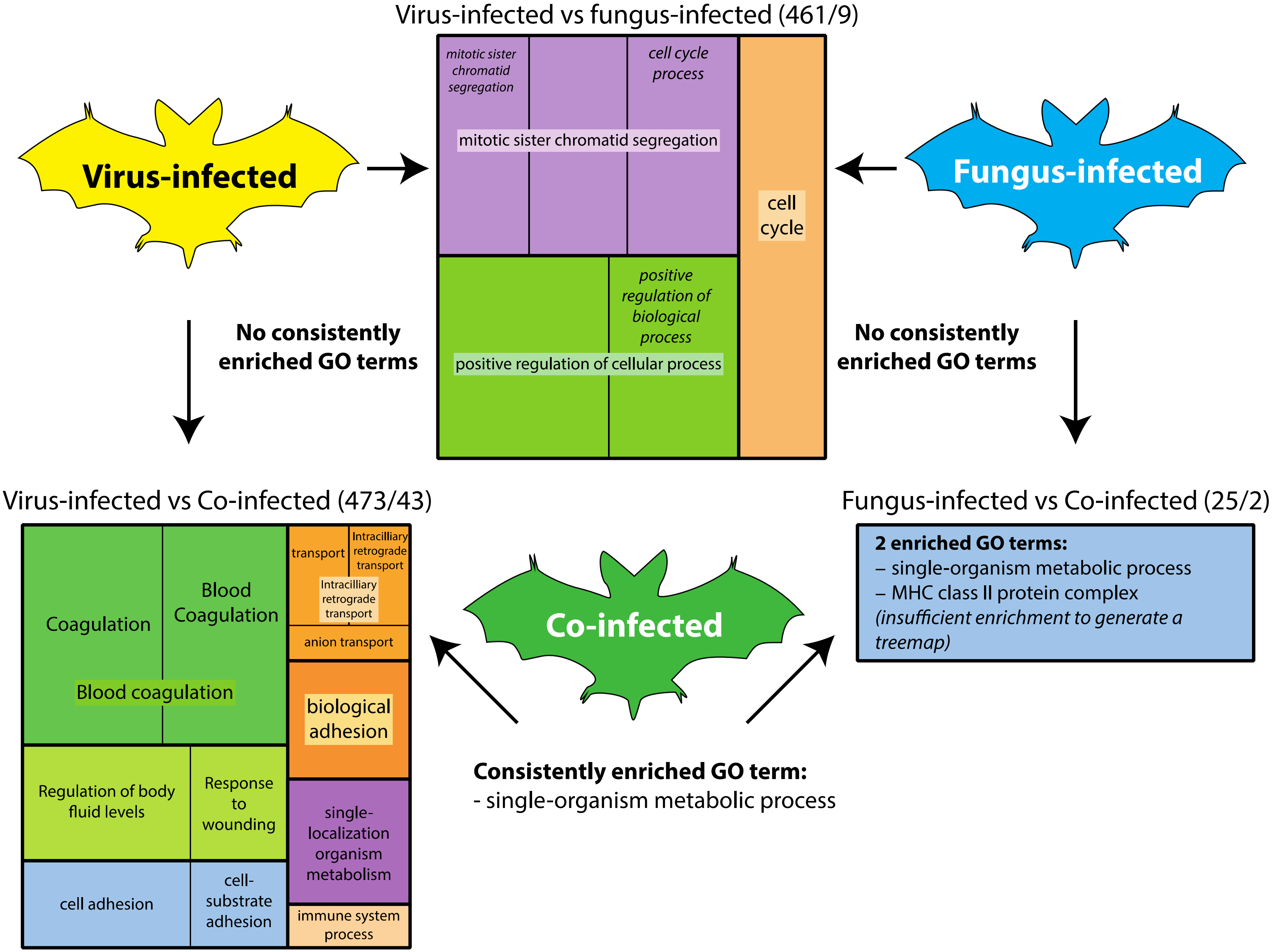

List of supplementary tables in the Dataset file:

Table S1. Read statistics of RNA-Seq samples.
Table S2. Differentially expressed genes determined by DESeq2 for the Negative (mock-infected) vs. MylCoV comparison.
Table S3. Differentially expressed genes determined by DESeq2 for the Negative (mock-infected) vs. *P. destructans* comparison.
Table S4. Differentially expressed genes determined by DESeq2 for the Negative (mock-infected) vs. Coinfected comparison.
Table S5. Differentially expressed genes determined by DESeq2 for the MylCoV vs. *P. destructans* comparison.
Table S6. Differentially expressed genes determined by DESeq2 for the Coinfected vs. MylCoV comparison.
Table S7. Differentially expressed genes determined by DESeq2 for the Coinfected vs. *P. destructans* comparison.
Table S8. Gene Ontology Enrichment Analysis (g:GOSt) summary using DESeq2 determined differentially expressed genes (>2 fold change, FDR<0.05).
Table S9. Details of primers used for PCR
